# The venom gland transcriptome of the parasitoid wasp *Nasonia vitripennis* highlights the importance of novel genes in venom function

**DOI:** 10.1186/s12864-016-2924-7

**Published:** 2016-08-08

**Authors:** Andre D. Sim, David Wheeler

**Affiliations:** Institute of Fundamental Sciences, Massey University, Palmerston North, 4442 New Zealand

**Keywords:** Ovary, RNA-seq, Transcriptomics, Serine protease, Drug development, Venom gene

## Abstract

**Background:**

Prior to egg laying the parasitoid wasp *Nasonia vitripennis* envenomates its pupal host with a complex mixture of venom peptides. This venom induces several dramatic changes in the host, including developmental arrest, immunosuppression, and altered metabolism. The diverse and potent bioactivity of *N. vitripennis* venom provides opportunities for the development of novel acting pharmaceuticals based on these molecules. However, currently very little is known about the specific functions of individual venom peptides or what mechanisms underlie the hosts response to envenomation. Many of the venom peptides also lack bioinformatically derived annotations because no homologs can be identified in the sequences databases. The RNA interference system of *N. vitripennis* provides a method for functional characterisation of venom protein encoding genes, however working with the current list of 79 candidates represents a daunting task. For this reason we were interested in determining the expression levels of venom encoding genes in the venom gland, as this information could be used to rank candidates for further study. To do this we carried out deep transcriptome sequencing of the venom gland and ovary tissue and used RNA-seq to rank the venom protein encoding genes by expression level. The generation of a specific venom gland transcriptome dataset also provides further opportunities to investigate novel features of this specialised organ.

**Results:**

RNA-seq revealed that the highest expressed venom encoding gene in the venom gland was ‘Venom protein Y’. The highest expressed annotated gene in this tissue was serine protease *Nasvi2EG007167*, which has previously been implicated in the apoptotic activity of *N. vitripennis* venom. As expected the RNA-seq confirmed that venom encoding genes are almost exclusively expressed in the venom gland relative to the neighbouring ovary tissue. Novel genes appear to perform key roles in *N. vitripennis* venom function, with over half of the 15 highest expressed venom encoding loci lacking bioinformatic annotations. The high throughput sequencing data also provided evidence for the existence of an additional 472 previously undescribed transcribed regions in the *N. vitripennis* genome. Finally, metatranscriptomic analysis of the venom gland transcriptome finds little evidence for the role of *Wolbachia* in the venom system.

**Conclusions:**

The expression level information provided here for the *N. vitripennis* venom protein encoding genes represents a valuable dataset that can be used by the research community to rank candidates for further functional characterisation. These candidates represent bioactive peptides valuable in the development of new pharmaceuticals.

**Electronic supplementary material:**

The online version of this article (doi:10.1186/s12864-016-2924-7) contains supplementary material, which is available to authorized users.

## Background

*Nasonia vitripennis* (Hymenoptera: Pteromalidae) (here after *Nasonia*) is a parasitoid wasp that utilises species of cyclorrhaphous diptera, such as house flies and blow flies, as hosts to support the growth and development of their offspring. Envenomation of the host increases the overall nutritional value of the food source available to the feeding wasp larvae. Some of these venom induced changes include developmental arrest, immune suppression and changes to the overall metabolite profile of the host [1–3]. The diverse and potent bioactivities of *Nasonia* venom peptides indicate that they may be useful in the development of novel pharmaceuticals [1, 4–7]. The venom peptides themselves are synthesised in the venom gland (VG) before being secreted into the venom reservoir ready for injection into the host via the ovipositor [8]. A recent proteomic study on these venom reservoir extracts was able to identify 79 peptides [4, 9]. Although several of the peptides had similarity to sequences found in the venom of other animals, a majority had either not been associated with venom before or had no homology to sequences in the National Centre for Bioinformatic Information (NCBI) databases. Bioinformatic based annotation categories that could be applied to the venom peptides included immunity, metabolism, esterases, proteases, and recognition proteins [9]. The most prevalent functional annotation was serine proteases, which is one of the largest gene families found in insect genomes [4, 10].

The response to *Nasonia* envenomation by the common laboratory host *Sarcophaga bullata* (Diptera: Sarcophagidae) has been extensively studied at both the molecular and physiological level. Experiments show that the respiration rate of *S. bullata* remains stable for up to 2 weeks following envenomation (although at lower levels than unstung hosts) indicating that the venom is not immediately lethal to this host [11]. Other physiological changes to the host associated with envenomation include an increase in lipids and free amino acids, as well as the initiation of developmental arrest through an as yet an unknown mechanism. *S. bullata* immune processes are also affected, with the abundance and potency of circulating plasmatocytes declining dramatically (due to cell death) 60 min following envenomation by *Nasonia* [12]. Importantly, *Nasonia* venom has been shown to be able to interfere with mammalian immunity pathways. For example, experiments using mouse cell lines demonstrated that *Nasonia* venom has both antimicrobial and anti-inflammatory properties [6]. Reporter assays provided evidence that the observed immune responses were mediated through venom specific modifications to the steroid and Toll pathways [6]. The activity of *Nasonia* venom in mammalian systems may indicate that venom peptides have evolved to target highly conserved core pathway components in order to limit the ability of the host to escape envenomation. In this situation evolution has already done much of the work in designing highly specialised peptides that target cellular pathways involved in diseases such as hypertension, diabetes, and cancer. Thus given the incredible species richness of the parasitoid wasps their venoms may represent a diverse untapped reservoir of molecules ready for incorporation into drug development pipelines.

In contrast to the extensive body of work exploring the host response to envenomation, relatively little is known about the individual venom peptides responsible for inducing these changes. Based on sequence homology a *Nasonia* venom metalloproteinase has been proposed to initiate developmental arrest by interfering with Notch signalling [1, 7, 13]. Supporting this proposal is the observation that several notch pathway genes are differentially expressed in the *S. bullata* hosts following envenomation. However, currently there is no direct evidence showing that this venom metalloprotease is specifically responsible for the observed expression changes in the Notch pathway [1]. Several other venom peptides are thought to be involved in modifying host metabolic pathways based on homology to sequences from model systems. For example, a venom lipase could be responsible for the increase in phospholipid degradation observed in envenomated hosts [2, 14]. A venom trehalase has been proposed to convert the abundant host trehalose sugars into glucose [2]. Additionally, several proteases found in *Nasonia* venom are homologous to peptides used by the ectoparasitoid *Euplectrus separatae* and the tick *Haemaphysalis longicornis* for blood meal digestion [15, 16].

The most direct functional characterisation of a venom encoding gene comes from a recent RNA interference (RNAi) study by Siebert et al. (2015). Knockdown of *Nasonia* venom calreticulin (*Nasvi2EG037342*) resulted in an increase in melanisation occurring at the oviposition wound on the host following envenomation [5]. This observation suggested that venom calreticulin functions to interfere with normal wound healing in the host, perhaps by competing with the endogenous calreticulin in the host for binding of cofactors [17].

Several endoparasitoids from the Braconidae and Ichneumonidae wasp families inject viruses or virus like particles along with their venom in order to prevent the host immune system from attacking eggs deposited in the haemolymph. The virus like particles appear to provide protection to the developing parasitoid by induction apoptosis in host hemocytes [18–22]. As yet viral-like particles have not been implicated in *Nasonia* venom function and viral transcripts could not be identified in cDNA libraries synthesised from envenomated hosts [23]. The intracellular symbiont *Wolbachia* is also present in high concentrations in the ovaries and host envenomation has been proposed as a possible mechanism for horizontal transfer of strains between parasitoid species [24].

Currently there is little direct experimental evidence supporting the proposed functions of the 79 *Nasonia* venom proteins and many of the bioinformatic annotations are based on homology to sequences from non-venomous model organisms. The RNAi knockdown system of *Nasonia* clearly provides an opportunity to begin to functionally characterise the venom protein encoding genes [5]. However, given the number of genes involved, an important first step would be the identification of high value candidates. For this reason we carried out deep sequencing of the *Nasonia* VG and ovary in order to use the relative gene expression as a proxy for protein abundance in the venom reservoir. The RNA-seq information generated in this study also provides a valuable dataset for future studies into the reproductive system of this important developing model system.

## Results

### RNA-seq analysis of the *Nasonia* VG and ovary transcriptomes

**Fig. 1:**
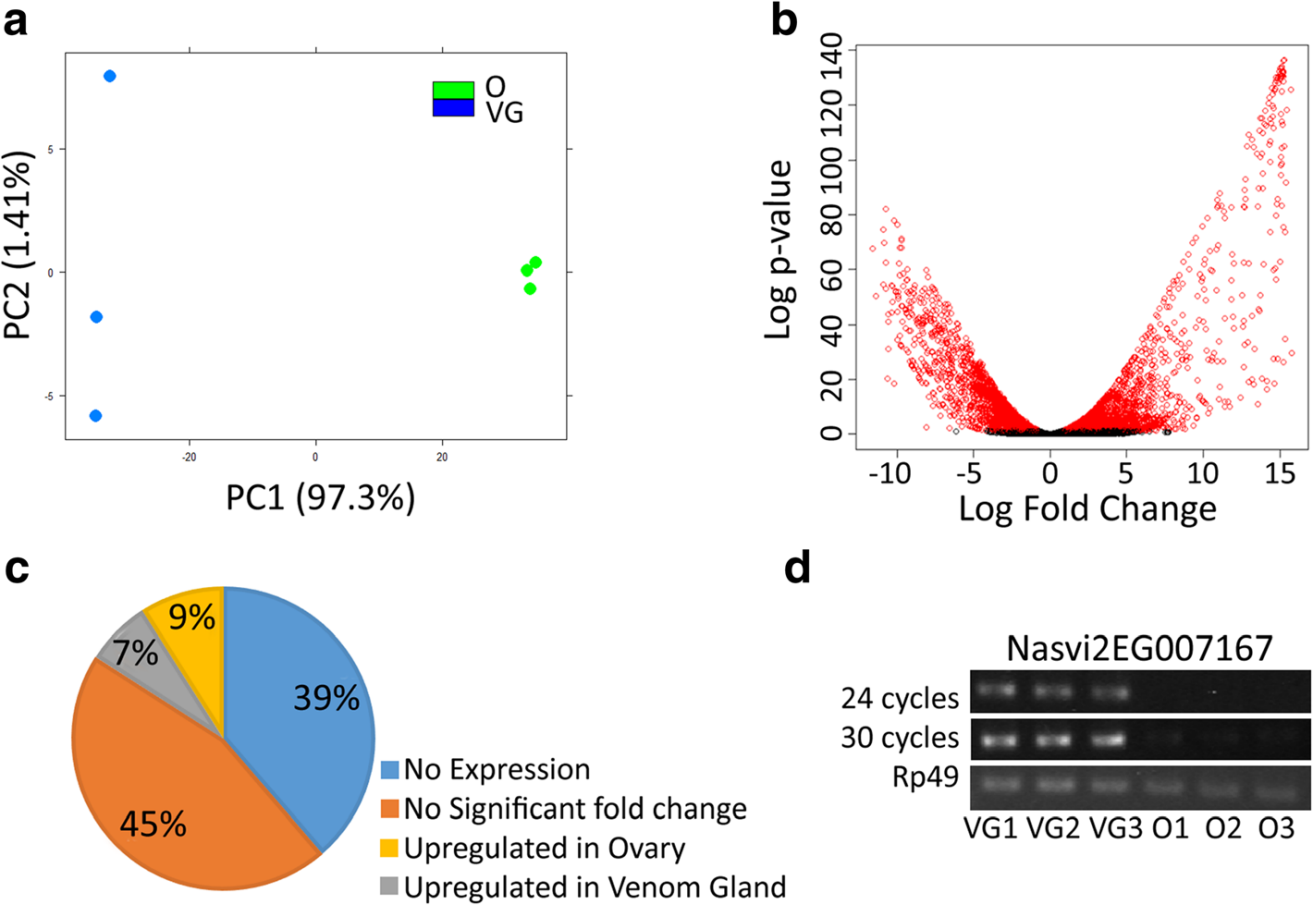
RNA-seq highlights expression differences between ovary and VG tissue. **a** Principal component analysis of expression profiles for ovary (*green*) and VG (*blue*) replicates showing the samples clustering by treatment group. The percentage on the axis labels represents the total variance explained by that component (**b**) Volcano plot of the RNA-seq data with positive fold change representing up regulated genes in the VG and vice versa for ovary. Genes coloured as red were differentially expressed at an adjusted p-value < 0.05. **c** Pie chart summarising the proportion of genes that were part of each expression category (adjusted *p*-value < 0.05). **d** Semi-quantitative RT-PCR targeting the highest expressed annotated venom encoding gene *Nasvi2EG007167*. Amplification of *Nasvi2EG007167* cDNA could be detected at 24 cycles in the venom gland cDNA replicates, whilst only a faint band could be detected at 30 cycles for the ovary samples. The house-keeping gene RP49 was used as a loading control. VG1-3 and O1-3 represent independent replicates for venom gland and ovary samples, respectively

We used RNA-seq to measure the expression of venom protein encoding genes in the VG and ovary. We chose the ovary as the comparative tissue because although it is connected to the venom apparatus, it clearly performs a distinct function in *Nasonia* reproduction. High throughput sequencing of VG and ovary RNA-seq libraries (three biological replicates each tissue) generated a total of 92.7 million and 97.9 million of high quality reads (phred quality score >20), respectively. On average 90.7 % of the sequenced reads successfully mapped to the published *Nasonia* genome [4] (Table 1). The lower mapping percentages observed for VG sample three (“VG 3” in Table 1) was due to a higher proportion of rRNA reads in this sample. We predict that this resulted from less effective rRNA removal during library preparation for this replicate. Apart from the higher proportion of *Nasonia* rRNA reads other quality control parameters for this replicate were consistent with those obtained from the other samples (Fig. 1a and Table 1).

**Table 1:** RNA-seq statistics of *Nasonia vitripennis* ovary and VG tissue

Sample	Total Reads	Mapped (%)	Assigned (%)^a^	Multiple Alignments (%)^b^	No Feature (%)^c^
VG 1	2.89E + 07	95.62	88.08	3.63	1.99
VG 2	3.24E + 07	96.15	87.31	3.37	1.97
VG 3	3.14E + 07	67.03	59.65	3.54	4.32
Ovary 1	3.31E + 07	95.17	85.51	1.43	5.95
Ovary 2	3.42E + 07	95.16	84.06	1.46	6.34
Ovary 3	3.06E + 07	95.16	84.18	1.43	6.13

^a^Proportion of reads which could be aligned to a gene model

^b^Proportion of reads assigned to multiple gene models

^c^Proportion of aligned reads that did not overlap a gene model

Overlap between the mapped reads and annotated features in the *Nasonia* official gene set version 2 (OGS2) is high, with an average of 81 % of mapped reads aligning to at least one gene body (Table 1). Of the 36,329 annotated genes in OGS2, 54 % (19,854) had detectable expression in either the VG or ovary. Based on normalised read counts, tissue specific expression (reads for a gene model detected in only one tissue) was observed for 3475 genes in the ovary and 1424 genes in the VG. Next we utilised the R-package DESeq to identify differentially expressed genes between the VG and ovary RNA-seq datasets [25]. Significantly different expression was observed for 5794 (16 % of OGS2 loci) genes at an adjusted *p*-value threshold of 0.05 (Benjamini-Hochberg adjusted for multiple testing) (Figs. 1b and c; Additional file 1). Of the differentially expressed genes, 2524 were up and 3270 were down regulated in the VG relative to the ovary. Overall the fold changes of upregulated genes were significantly larger in the VG versus the ovary (Student’s *t*-test *p*-value < 0.001) (Fig. 1b).

**Fig. 2:**
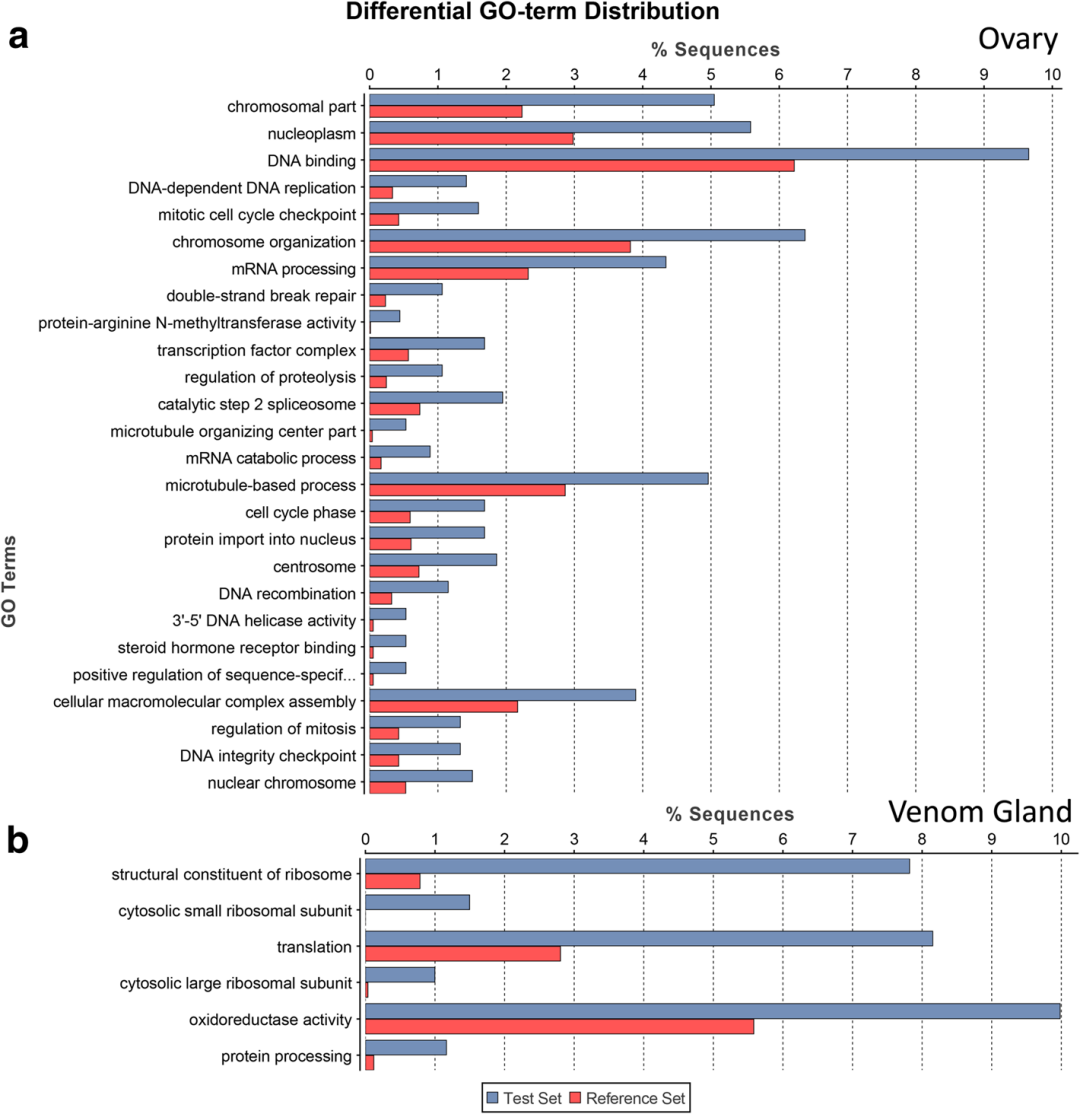
GO terms related to ribosomal function, translation, and protein processing are enriched in the VG. Enriched GO terms amongst the subset of genes significantly upregulated in the ovary (**a**) and VG (**b**). The bar graph represents the proportion (in percentage) of sequences in the differentially expressed subset (*blue*) with the indicated functional annotation, compared to the proportion of these annotations assigned to the entire gene set (*red*)

Using Blast2GO we then looked for GO term enrichment amongst the genes either significantly upregulated in the ovary or VG tissues. The reproductive function of the ovary is characterised by several GO terms related to cell division and transcription (Fig. 2a). In contrast, genes significantly upregulated in the VG showed GO enrichment in terms related to translation and protein processing functions (Fig. 2b). The relatively narrow GO functional categorisations of genes upregulated in the VG are likely to reflect that the major model systems are non-venomous.

### The highly expressed venom protein encoding genes

**Fig. 3:**
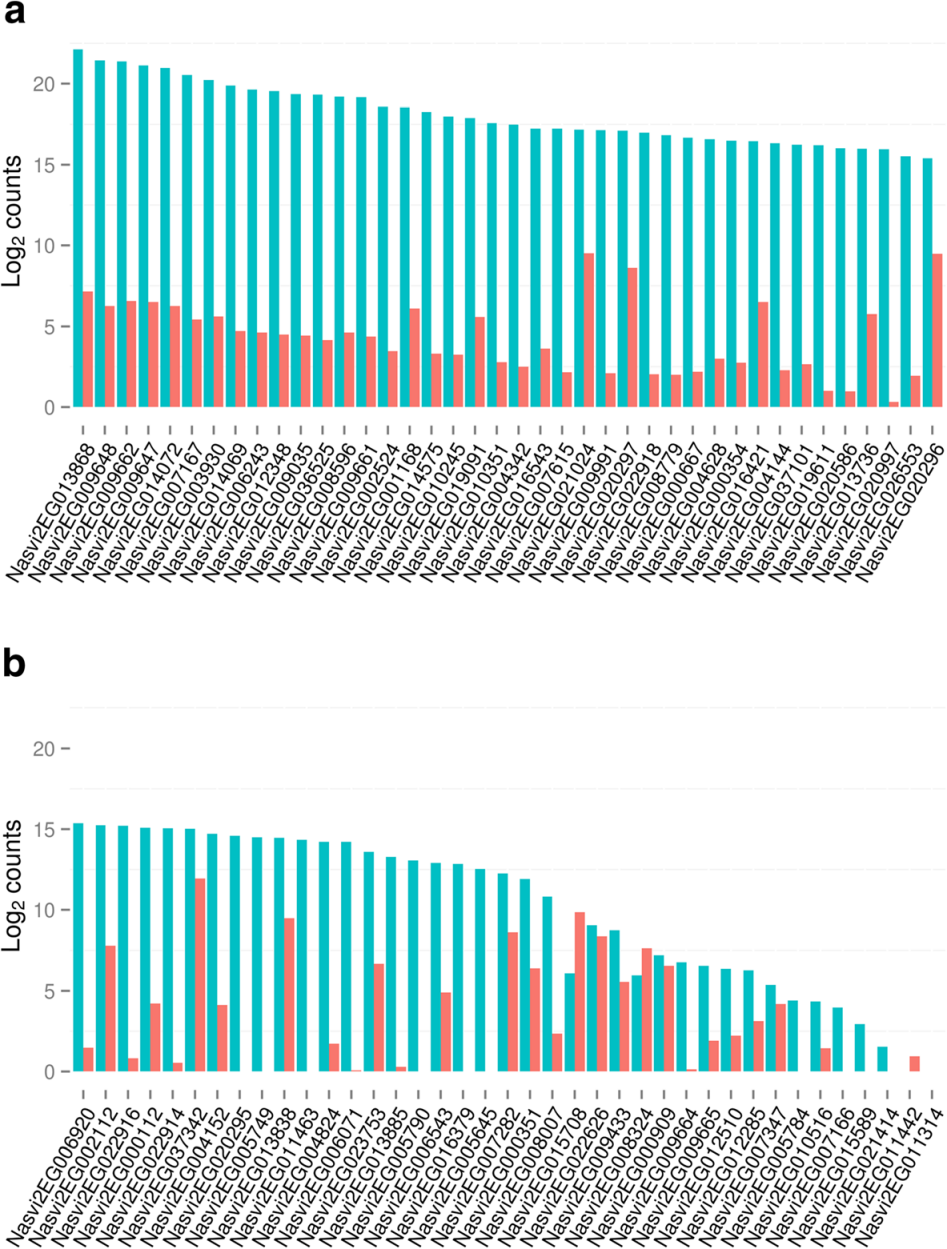
Venom genes were highly expressed in the VG. Log_2_ normalised gene counts for the 40 highest expressed (**a**), and 39 lowest expressed (**b**), venom protein encoding genes (sorted by highest observed loci count). VG counts are shown in blue and ovary counts in orange

Previous proteomic studies on *Nasonia* venom reservoir extracts identified 79 venom peptides. Based on our VG RNA-seq data we were able to detect expression from all but one of these venom protein encoding genes (Table 2). *Nasonia* venom genes were significantly more likely to be expressed at higher levels in the VG relative to the ovary (70 of 79 genes; Chi-square *p*-value < 0.0001 with Yates correction). The DESeq analysis showed that six of the venom protein encoding genes were expressed at equal levels in the VG and ovary (Table 2). Two venom protein encoding genes expressed at higher levels in the ovary than the VG were a aspartylglucosaminidase (*Nasvi2EG015708*) and trehalase (*Nasvi2EG008324*). The highest expressed venom encoding gene based on normalised venom gland read counts was the novel gene Venom protein Y (Nasvi2EG013868). A trypsin-like serine protease (*Nasvi2EG007167*) was the highest expressed annotated gene in the VG (Table 3). The RNA-seq data and semi-quantitative PCR showed that expression of *Nasvi2EG007167* was highly tissue specific (Fig. 1d). Only five of the top 15 highest expressed venom protein encoding genes had functional annotations, highlighting the unique functional properties of *Nasonia* venom (Tables 2 and 3). Next we used sequence homology based clustering to identify any relationships amongst the highly expressed unannotated genes. Using this approach only two small clusters of unannotated venom encoding genes were identified (V/Z and L/K), suggesting that most of these novel loci have evolved independently, rather than through an expansion of a single gene cluster (Additional file 2). Calreticulin (*Nasvi2EG037342*) was the highest ovary expressed venom encoding gene, which is not surprising given it also functions as a core immune response gene [26–28]. The single venom encoding gene that was not expressed in our venom dataset, a trypsin-1 serine protease (*Nasvi2EG011442*), also had very little expression in the ovary (Fig. 3 and Table 2).

**Table 2:** Summary of VG and ovary RNA-seq data for the 79 *Nasonia* venom protein encoding genes

	Gene ID	Ovary read counts^b^	VG read counts^b^	Log_2_ Fold Change^c^	Adj p-val	Classification
High^a^	Nasvi2EG013868	141	4600307	15.00	2.33E-127	Venom protein Y
	Nasvi2EG009648	76	2859048	15.20	7.49E-129	Venom protein G
	Nasvi2EG009662	93	2701601	14.82	7.75E-126	Venom protein Z
	Nasvi2EG009647	90	2310849	14.65	2.5E-124	Venom protein Q
	Nasvi2EG014072	75	2063715	14.75	4.41E-85	Venom protein X
	Nasvi2EG007167	43	1532232	15.11	7.49E-129	Serine protease
Medium^a^	Nasvi2EG003930	49	1218607	14.61	5.88E-123	Serine protease inhibitor 2
	Nasvi2EG014069	26	976928	15.20	1.2E-106	Venom protein L
	Nasvi2EG006243	25	819734	15.03	5.88E-111	γ-glutamyltranspeptidase
	Nasvi2EG012348	23	756942	15.04	1.14E-125	Venom protein H
	Nasvi2EG009035	22	666528	14.92	5.13E-127	Endonuclease-like
	Nasvi2EG036525	18	660159	15.17	4.8E-129	Aminotransferase-like
	Nasvi2EG008596	25	595426	14.56	7.8E-121	Venom protein P
	Nasvi2EG009661	21	590933	14.80	1.37E-124	Venom protein V
	Nasvi2EG002524	11	386873	15.11	1.45E-74	Venom protein I
	Nasvi2EG001168	68	385586	12.47	3.96E-92	Immunoglobulin-like
	Nasvi2EG014575	10	312136	14.94	1.22E-99	Venom protein K
	Nasvi2EG010245	10	257730	14.72	4.26E-84	Odorant-binding protein
	Nasvi2EG019091	48	245164	12.31	4.4E-13	Venom protein M
	Nasvi2EG010351	7	197286	14.79	1.09E-124	Glucose dehydrogenase-like
	Nasvi2EG004342	6	181642	14.97	9.19E-125	Lipase-like
	Nasvi2EG016543	12	155729	13.63	3.31E-110	Venom protein N
	Nasvi2EG007615	4	153498	15.08	1.03E-125	Lipase
	Nasvi2EG021024	737	149042	7.66	3.33E-50	Serine protease homologue
	Nasvi2EG009991	4	145328	15.06	3.41E-127	Acid phosphatase
	Nasvi2EG020297	387	141654	8.52	6.11E-59	Serine protease/CLIP
	Nasvi2EG022918	4	130887	14.97	7.1E-125	Serine protease
	Nasvi2EG008779	4	115742	14.82	1.21E-124	Aminotransferase-like
	Nasvi2EG000667	5	104214	14.48	1.04E-119	Serine proteinase inhibitor
	Nasvi2EG004628	8	98159	13.59	4.58E-65	Venom protein D
	Nasvi2EG000354	7	91416	13.72	2.06E-19	Cysteine-rich/TIL 1
	Nasvi2EG016421	91	90433	9.96	9.19E-65	α-Esterase
	Nasvi2EG004144	5	81771	14.02	6.13E-19	Venom protein T
	Nasvi2EG037101	6	77272	13.57	2.54E-48	Chitin binding protein-like
	Nasvi2EG019611	2	75339	15.19	5.42E-125	Laccase
	Nasvi2EG020586	2	66726	15.06	9.42E-120	Serine protease
	Nasvi2EG013736	54	64975	10.24	2.62E-45	Serine protease inhibitor 1
	Nasvi2EG020997	1	63251	15.64	7.83E-121	Venom protein J
	Nasvi2EG026553	4	46698	13.56	3.89E-109	Lipoprotein receptor-like
	Nasvi2EG020296	716	42847	5.90	1.43E-32	Serine protease/CLIP
	Nasvi2EG006920	3	42299	13.91	9.42E-113	Arylsulphatase b
	Nasvi2EG002112	224	38349	7.42	1.67E-47	β-1,3-Glucan recognition
	Nasvi2EG022916	2	38281	14.41	4.45E-79	Serine protease
	Nasvi2EG000112	18	34599	10.87	3.97E-83	Apyrase
	Nasvi2EG022914	1	33717	14.50	1.78E-111	Serine protease
	Nasvi2EG037342	3926	33363	3.09	3.21E-04	Calreticulin
	Nasvi2EG004152	18	26602	10.57	2.07E-79	Venom protein R
	Nasvi2EG020295	1	24624	14.74	1.28E-113	Serine protease/CLIP
	Nasvi2EG005749	1	23005	14.93	1.58E-59	Serine protease
	Nasvi2EG013838	711	22562	4.99	5.35E-11	Venom protein F
	Nasvi2EG011463	1	20535	14.43	9.18E-110	Inositol phosphatase
	Nasvi2EG004824	3	19029	12.49	6.8E-51	Venom protein E
Low^a^	Nasvi2EG006071	1	18923	14.14	9.03E-97	Venom protein S
	Nasvi2EG023753	103	12408	6.91	3.68E-42	Dipeptidylpeptidase IV
	Nasvi2EG013885	1	9901	13.00	2.6E-102	γ-Glutamyl transpeptidase 2
	Nasvi2EG005790	1	8481	13.24	6.37E-25	Antigen-5 like
	Nasvi2EG006543	30	7665	8.00	2.54E-40	γ-Glutamyl cyclotransferase-
	Nasvi2EG016379	0	7408	15.24	3.96E-78	Venom protein U
	Nasvi2EG005645	0	5919	13.75	5.86E-31	Venom protein O
	Nasvi2EG007282	390	4909	3.65	1.54E-09	Serine protease
	Nasvi2EG000351	84	3807	5.51	1.44E-01	Cysteine-rich/TIL 2
	Nasvi2EG008007	5	1805	8.46	3.43E-27	Angiotensin-converting
	Nasvi2EG022626	326	532	0.71	6.18E-01	Serine protease/CUB
	Nasvi2EG009433	47	426	3.17	1.90E-04	Cysteine-rich/KU
	Nasvi2EG000909	93	147	0.66	4.82E-01	C1q-like venom protein
	Nasvi2EG009664	1	110	6.65	1.19E-03	Cysteine-rich/Pacifastin 1
	Nasvi2EG009665	4	93	4.62	2.44E-03	Cysteine-rich/Pacifastin 2
	Nasvi2EG012510	5	83	4.17	1.23E-04	Chitinase 5
	Nasvi2EG012285	9	77	3.15	1.94E-05	Acid phosphatase
	Nasvi2EG015708	929	68	−3.76	1.14E-15	Aspartylglucosaminidase
	Nasvi2EG008324	200	62	−1.70	4.03E-04	Trehalase
	Nasvi2EG007347	18	41	1.19	1.00E + 00	Serine protease
	Nasvi2EG005784	1	21	4.63	3.49E-02	Antigen 5-like protein
	Nasvi2EG010516	3	20	2.88	1.03E-02	Metalloprotease
	Nasvi2EG007166	0	16	N/A	4.85E-06	Serine protease
	Nasvi2EG015589	1	8	3.37	7.42E-03	Laccase
	Nasvi2EG021414	0	3	4.23	3.08E-01	Nucleoside hydrolase
	Nasvi2EG011314	0	1	N/A	7.67E-01	Venom protein W
	Nasvi2EG011442	1	0	N/A	1.00E + 00	Serine protease

^a^Expression category, see methods for details

^b^Library size normalised read counts from DESeq divided by transcript length (in kb) and sorted in descending order based on the venom gland values

^c^Fold changes calculated directly from library size normalised read counts by DESeq

**Table 3:** Highest 15 expressed genes in the VG and ovary sorted in descending order by normalised read counts

Venom gland data					
Gene ID	Ovary normalised counts^b^	VG normalised counts^b^	Log_2_ Fold Change^c^	Adj p-val	Classification
Nasvi2EG013868^a^	141	4600307	15.00	2.33E-127	Venom protein Y
Nasvi2EG009648^a^	76	2859048	15.20	7.49E-129	Venom protein G
Nasvi2EG009662^a^	93	2701601	14.82	7.75E-126	Venom protein Z
Nasvi2EG009647^a^	90	2310849	14.65	2.50E-124	Venom protein Q
Nasvi2EG014072^a^	75	2063715	14.75	4.41E-85	Venom protein X
Nasvi2EG021198	62	1557446	14.61	6.16E-55	Unknown
Nasvi2EG007167^a^	43	1532232	15.11	7.49E-129	Serine Protease
Nasvi2EG003930^a^	49	1218607	14.61	5.88E-123	Serine protease inhibitor 2
Nasvi2EG009363	54	1217247	14.45	5.14E-121	Unknown
Nasvi2EG014069^a^	26	976927	15.20	1.20E-106	Venom protein L
Nasvi2EG006243^a^	25	819734	15.03	5.88E-111	γ-glutamyltranspeptidase
Nasvi2EG012348^a^	23	756942	15.04	1.14E-125	Venom protein H
Nasvi2EG011080	20	722740	15.15	3.50E-126	Unknown
Nasvi2EG009035^a^	22	666528	14.92	5.13E-127	Endonuclease-like
Nasvi2EG036525^a^	18	660159	15.17	4.80E-129	Aminotransferase-like 2
Ovary data					
Nasvi2EG004175	35984	37	−9.92	4.96E-65	Unknown
Nasvi2EG002830	34980	10187	−1.78	7.40E-05	HEAT shock 70
Nasvi2EG000341	29283	27085	−0.11	1	CCHC-type zinc finger
Nasvi2EG007353	29093	708.41	−5.36	2.33E-32	ATP-dependent RNA helicase
Nasvi2EG002390	25623	73530	1.52	2.81E-3	40S ribosomal protein
Nasvi2EG006904	25600	45210	0.82	2.70 E-1	Elongation factor 1-alpha
Nasvi2EG011214	25321	70701	1.48	2.69E-2	40S ribosomal protein
Nasvi2EG020214	25158	139912	2.48	1.38E-4	60S acidic ribosomal protein
Nasvi2EG004598	25153	72614	1.53	2.70E-3	60S ribosomal protein
Nasvi2EG007380	23250	117959	2.34	1.28E-3	60S acidic ribosomal protein
Nasvi2EG019818	23062	85908	1.90	1.68E-4	40S ribosomal protein
Nasvi2EG003839	22711	103851	2.20	1.39E-05	60S ribosomal protein
Nasvi2EG007413	22602	92995	4.11	5.36E-05	40S ribosomal protein
Nasvi2EG011575	21836	57655	2.64	2.21E-2	60S ribosomal protein
Nasvi2EG014261	21805	219749	10.08	3.29E-11	Unknown

^a^Indicates venom protein encoding gene

^b^Library size normalised read counts from DESeq, divided by transcript length (in kb) and sorted in descending order based on the venom gland values

^c^Fold changes calculated from library size normalised read counts by DESeq

### RNA-seq identifies 472 novel genes

**Fig. 4:**
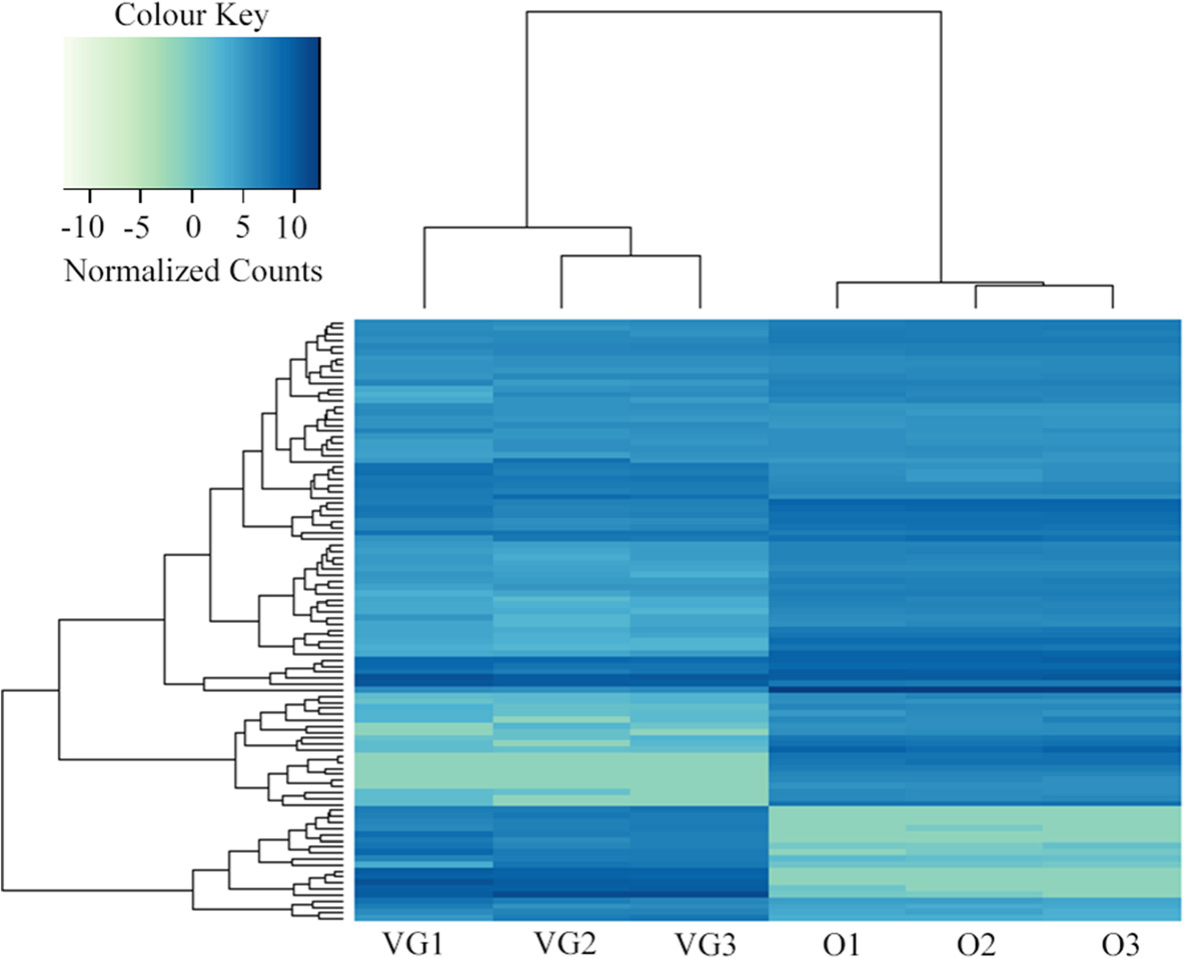
Heatmap of normalised counts for the 100 highest expressed novel genes identified by cufflinks in the ovary and VG expression data. The top 100 novel genes identified by cufflinks based on mean expression in the VG and Ovary colour coded by expression level. VG1-3 and O1-3 represent data from the three VG and ovary replicates, respectively. The dendrogram shows the clustering of samples based on euclidian distance

**Fig. 5:**
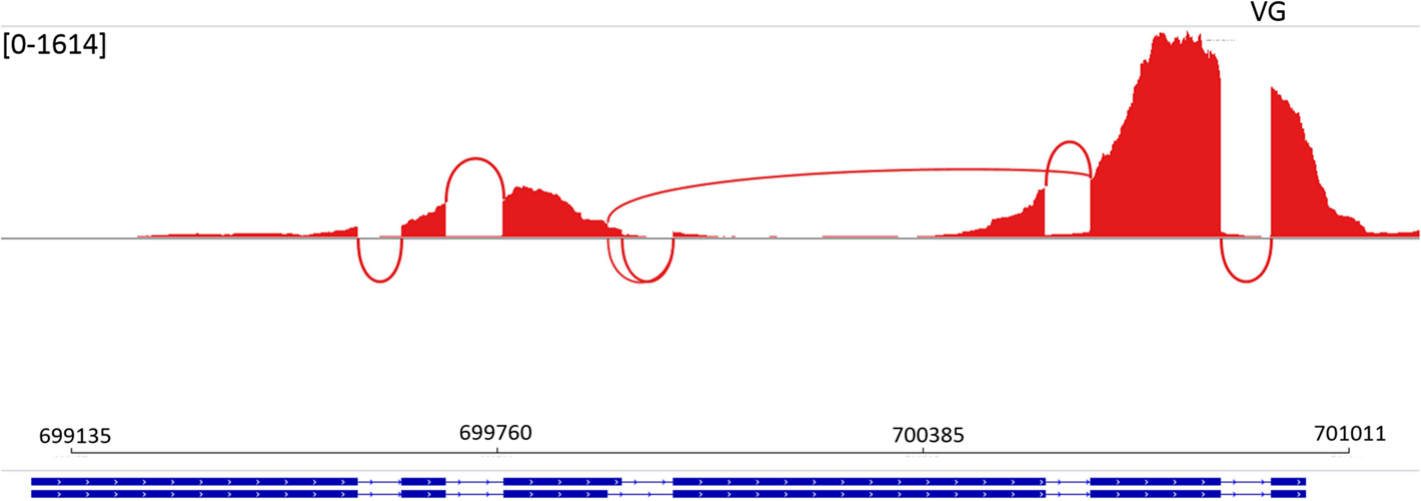
Differential splicing of VG specific novel gene XLOC_013208. The coverage of mapped reads over the two predicted gene models for XLOC_013208 the highest expressed novel gene in the VG. The coverage range is displayed in the square brackets in the top left corner of the figure. The blue bar represents the gene model as predicted by cufflinks. The thick sections represent exons and the thin lines represent introns. The rightward pointing arrows represent the orientation of the gene and the numbers above are for reference to the base pair position on scaffold 29

The generation of the official gene set for *Nasonia* did not include evidential sequencing data specifically obtained from VG samples, so we predicted that genes expressed in this relatively small organ might be underrepresented in the current gene models. Therefore, we used the Cufflinks software package to identify novel genes based on mapped reads that did not overlap with any current annotations in OGS2 [29] (Additional file 3). This approach identified 472 putative genes from both VG and ovary datasets (Additional file 4). We merged these new gene models into the official gene set and repeated the DESeq differential expression analysis (Additional file 5). This showed that 140 (30 %) of the novel genes were significantly upregulated and 56 (12 %) were significantly down regulated in the VG tissue; the remaining 276 genes (58 %) were not differentially expressed between the tissues (Fig. 4). The highest expressed novel genes in both the VG and ovary were shown to be highly tissue specific. The most differentially expressed novel VG gene (based on fold change) XLOC_013208 was also predicted to have an alternate splice form that truncated exon 3 (Fig. 5). Unfortunately, the putative proteins encoded by XLOC_013208 splice forms have no homology to any domains in the database so we are not able to predict possible functional consequences of this splicing event. As a result of low homology to sequences in the NCBI database only ten of the 472 novel genes identified here could be annotated with gene ontology terms using Blast2GO (Table 4). Extending the Cufflinks splice form prediction methodology to the currently annotated venom protein encoding genes failed to identify any significant alternative splice forms between the VG and ovary transcriptomes.

**Table 4:** Gene annotations of novel detected transcripts from *Nasonia vitripennis* VG and Ovary samples

Gene ID	Classification	GO terms
XLOC_005019	Atp-dependent dna helicase q4	metal ion binding, helicase activity, heterocyclic compound binding, organic cyclic compound binding
XLOC_010355	Signal peptidase complex catalytic subunit sec11a	signal peptide processing, integral to membrane, proteolysis, serine-type peptidase activity
XLOC_012101	N-acetylneuraminate lyase-like	lyase activity, metabolic process
XLOC_013961	NADH dehydrogenase subunit 2	mitochondrial electron transport, NADH to ubiquinone, mitochondrial inner membrane, respiratory chain, respiratory chain, NADH dehydrogenase (ubiquinone) activity, integral to membrane
XLOC_015645	NADH dehydrogenase subunit partial	integral to membrane, mitochondrion, oxidation-reduction process, NADH dehydrogenase (ubiquinone) activity
XLOC_016115	Uncharacterized aarf domain-containing protein kinase 1	protein phosphorylation,oxidation-reduction process, protein kinase activity, ubiquinone biosynthetic process, ATP binding, flavin adenine dinucleotide binding oxidoreductase activity, acting on paired donors, with incorporation or reduction of molecular oxygen, NAD(P)H as one donor, and incorporation of one atom of oxygen
XLOC_016465	60s ribosomal protein	ribosome, structural constituent of ribosome, translation
XLOC_019886	28 s ribosomal protein mitochondrial	ribosome, structural constituent of ribosome, translation
XLOC_020689	signal peptidase complex subunit 3	peptidase activity, signal peptide processing, integral to membrane, signal peptidase complex
XLOC_022279	inosine-5 -monophosphate dehydrogenase 1b	oxidation-reduction process, adenyl nucleotide binding, metal ion binding, GMP biosynthetic process, cytoplasm, IMP dehydrogenase activity

### Expression of venom protein encoding genes previously identified as high value candidates

Using our data we were then able to examine the expression pattern of genes previously identified as high value venom candidates based on bioinformatic annotations or results from studies in other venomous species [7, 9]. To differentiate the genes based broadly on their expression level we placed the 79 venom protein encoding genes into high, medium or low expression categories based on three equal divisions of normalised counts across our entire dataset (see methods). These categories were defined by normalised read count ranges of <181700, 181701-1218700,>1218701 for low, medium and high, respectively.

The highly expressed category was dominated by novel venom genes (Venom proteins G, Q, X, Y, and Z), with the single annotated loci being the trypsin-like serine protease *Nasvi2EG007167* (Table 2). Serine proteases play broad functional roles across insect physiology, including apoptosis, immunity, and development [30, 31]. The medium expression category includes the gamma-glutamyltranspeptidase and endonuclease-like venom genes, which have been previously implicated in initiating venom induced apoptosis by interfering with normal metabolism of glutathione and by degrading nuclear DNA, respectively [7, 32, 33]. Similarly, the aminotransferase-like venom gene encodes an enzyme predicted to produce kynurenic acid from kynurenine and this may also be involved in apoptosis [34, 35]. Other medium expression category genes of note include IMPL-L2, enzymes involved in carbohydrate metabolism, and several serine proteases/proteinase inhibitor genes. Insulin binding protein (*Nasvi2EG001168*) is possibly involved in initiating the developmental arrest by blocking insulin signalling [7, 36].

The majority of venom protein encoding genes fit into the low expressed category based on our designations. Notable venom protein encoding genes in the low expression group include apyrase, beta-1-3-Glucan recognition protein, dipeptidyl peptidase, metalloprotease, trehalase, chitinase, and all five of the venom cystein-rich/Pacifastin protease inhibitors (Table 2). The first three of these aforementioned genes have been proposed to be involved in modifying the host immune response [7, 37, 38]. The metalloprotease has been suggested to be involved in venom induced developmental arrest by interfering with normal Notch signalling [1]. The trehalase gene important for metabolising carbohydrates is also one of the few such genes expressed at higher levels in the ovary in our dataset. As noted by [7] the observation of proteins normally associated with breakdown of the chitin rich cuticle is somewhat puzzling given *Nasonia* larvae are able to mechanically access the host hemolymph using their mandibles [7, 39]. Antigen 5-like protein and the cystein-rich/pacifastin protease inhibitors have been identified in the venom of the *Apis mellifera* and *Pimpla hypochondriaca* [40, 41].

### Metatranscriptomic analysis of *Nasonia* VG and ovary tissue

**Fig. 6:**
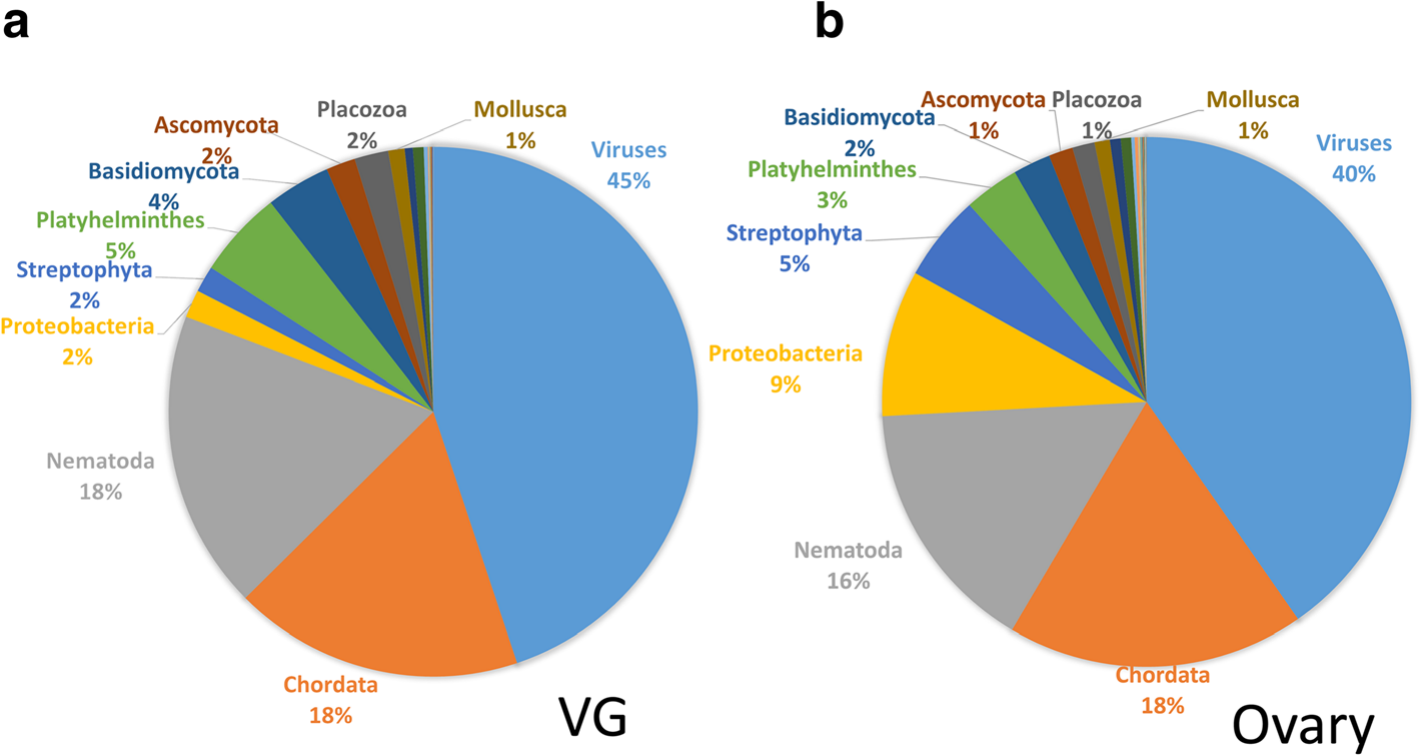
Differences in metatranscriptomic composition of the VG and ovary. Distribution of reads that did not map to the *Nasonia* genome assigned to the phylum level from the VG **a** and ovary **b** RNA-seq data by the program Megan

Finally, although the library preparation method included an mRNA enrichment step, we were interested in looking for any evidence for the involvement of bacteria (such as *Wolbachia*) or viruses in *Nasonia* venom function. Therefore, we performed a metatranscriptomic analyses of reads that did not align to the *Nasonia* genome [42, 43]. At the phylum level reads from both tissues could be assigned to viruses (42.5 %), Nematoda (17 %), Chordata (18 %) and Proteobacteria (5.5 %). A large proportion of the unmapped reads were assigned to viruses, with most of these being identified as an uncharacterised ‘Nasonia vitripennis virus’ (Taxonomic id #626355) (Fig. 6). The nematode reads were assigned to *Brugia malayi* and *Trichuris trichiura*, thus we initially suspected that this result may represent hits to the *Wolbachia* symbionts that are associated with both of these species [44]. However, subsequent megablast searches against the NCBI nr/nt database with these reads revealed both the Nematoda and Chordata groupings were based on low diversity rRNA sequences that were taxonomically uninformative.

**Fig. 7:**
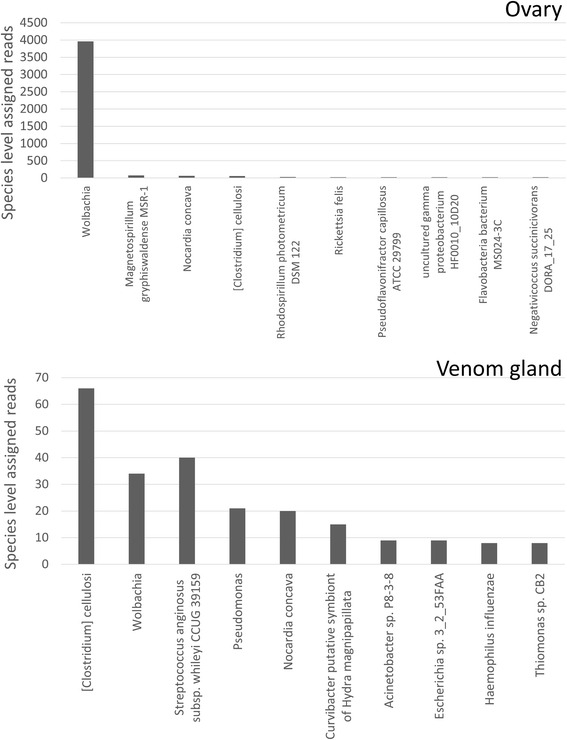
Proteobacteria species identified in the venom gland and ovary. Distribution of reads that did not map to the *Nasonia* genome, which can be assigned to Proteobacteria species in the ovary and VG data by Megan

As expected a large proportion (87 %) of the Proteobacteria reads from the ovary were assigned to *Wolbachia* species (Fig. 7). In contrast relatively few reads could be assigned to *Wolbachia* species in the Proteobacteria grouping from the VG. *Clostridium cellulosi* and *Streptococcus anginosus* were the most common species assignments in the VG, however, relative to the abundance of *Wolbachia* assigned reads in the ovary, very few reads could be assigned at this narrow taxonomic level in the VG data (Fig. 7). Thus based on this data there is little evidence supporting a role for microorganisms in *Nasonia* venom function.

## Discussion

The expression levels of venom protein encoding genes obtained in this study provides additional information that can be used to select candidates for further functional analysis using either RNAi or recombinant techniques. The ovary RNA-seq also provides a proxy for the tissue specificity of the venom encoding gene expression that can be used to further refine candidate selection. It should be noted that high venom gene expression does not automatically imply that the encoded peptide is responsible for important envenomation phenotypes. Other biological features, such as translational regulators, post-translational modifications and mRNA/protein stability could also be critical to the function of gene products in the venom context. Indeed, it has been argued that as a result of extensive posttranslational modifications, final venom protein composition is largely independent of transcriptional control [45, 46]. The legitimacy of this view has been challenged by a more recent study showing strong correlations between the venom transcriptomes and proteomes of several snake species [47]. Ultimately, additional high quality quantitative proteomic and transcriptomic data will be required to more fully appreciate the general importance of posttranslational mechanisms on venom composition. Finally, it is also worth considering that highly expressed venom genes may perform accessory functions, such as protecting the venom reservoir of the wasp from being attacked by its own venom, as has been observed in snakes [48].

The highest expressed annotated venom encoding gene was a trypsin-like serine protease. Unfortunately, serine proteases are a very common functional category in insect genomes making it difficult to propose a role for this highly expressed gene (especially in the venom context). Previous studies using Sf21 cells have shown that the apoptotic activity of *Nasonia* venom could be reduced by the addition of serine protease inhibitors [49]. Interestingly, amongst the ten most highly expressed venom protein encoding genes with annotations, all three point to roles in apoptosis [7, 32, 33]. This may highlight the importance of apoptosis for the early envenomation effects, perhaps by assisting with the distribution of venom throughout the host or by suppressing the immune system by destroying circulating hemocytes [49]. *Nasonia* venom has also been shown to induce apoptosis in *S. bullata* neural tissue and this has been suggested as a possible explanation for developmental arrest in envenomated this host [50].

Perhaps the most dramatic finding from this study was that over half of the top 15 expressed venom protein encoding genes have no functional annotation, including the highest expressed gene ‘Venom protein Y’. In retrospect maybe this should not be surprising given the complex evolutionary processes that drive specialisation of venom peptides [51]. However, this observation does highlight the value of candidate selection methods (such as RNA-seq) that are not biased towards genes with existing annotation information. Even for those genes that are bioinformatically annotated, it should be recognised that most of the functional data comes from experiments in model systems that are non-venomous.

Another interesting finding from this study is that several venom peptides that have been previously implicated in venom function were shown to be only lowly expressed in our data [1, 7, 9]. Perhaps the best example is the metalloprotease (*Nasvi2EG010516*) predicted to be involved in initiating developmental arrest, was one of the lowest expressed of the venom protein encoding genes in our dataset [1, 52]. We also observed that all five cysteine rich protease inhibitors suggested to be involved in disrupting host immunity by inactivating the pro-phenol oxidase cascade were also expressed at low levels [7]. The low expression of chitinase-type venom genes also suggests they may not be directly involved in venom function, especially given *Nasonia* larvae are able to mechanically disrupt the host integument during feeding.

Cufflink based transcript assembly of the VG and ovary RNA-seq data allowed us to identify 472 previously undescribed transcribed regions in the *Nasonia* genome. The low expression and lack of homology to other known genes might explain why they have not been previously described. Alternatively these observations may indicate they represent artefacts of the cufflinks methodology. Re-evaluation of the raw venom proteomic data may enable the identification of additional peptide fragments based on these new gene models. It is worth noting that the OGS2 models were developed based on RNA-seq data from the *Wolbachia* free Asymcx strain of *Nasonia.* As some of the new gene models identified here could be induced by *Wolbachia* infection they represent candidates for being involved in the cytoplasmic incompatibility phenotypes induced by this protobacteria.

Finally, the metatranscriptomic study revealed that viruses represented a large proportion of non-*Nasonia* reads in the VG and ovary transcripts. Particularly ‘Nasonia vitripennis virus’, which has a positive strand ssRNA genome and is part of the Picornavirales order and the Iflaviridae family but remains unassigned to lower taxonomic ranks [23]. ‘Nasonia vitripennis virus’ has a ssRNA genome so reads may be derived from either messenger RNA or the ssRNA genome. The latter, opens up the possibility of performing a *de novo* assembly of the viral genome from our transcriptome data. Studies have shown that this virus causes no observed detrimental effect on *Nasonia* and it remains an open question as to whether these transcripts end up in the venom itself [23]. As expected the ovary metatranscriptome was dominated by *Wolbachia* transcripts. In contrast, we identified relatively few *Wolbachia* sequences in the VG, suggesting this bacteria is unlikely to play an important role in venom function. However, it is important to note that the RNA-seq library preparation methods used in our study enriched for poly A+ reads and thus many bacterial sequences would not have been sequenced in this experiment. Follow up experiments using total RNA library preparation methods are required to conclusively rule out a possible role for *Wolbachia* in *Nasonia* venom function.

Animal venoms represent an important source of natural products with pharmaceutical value; as demonstrated by the development of important drugs, such as Exenatide from the Gila monster lizard, and Captopril isolated from the venom of the lancehead viper [53]. More recently molecules isolated from reptile and arthropod venom have been shown to have therapeutic value in the treatment of cancer [54, 55]. With this in mind the venom of parasitoid hymenoptera holds particular promise, given the incredible species richness of this group of animals, a potentially vast and unique source of molecules awaits discovery. We also predict that venoms target highly conserved core cellular pathways, as a mechanism to limit the ability of the host to evolve detoxification strategies. Indeed, *Nasonia* venom has been shown to have bioactivity against mammalian immune pathways, further highlighting the pharmaceutical value of these molecules [6]. The results reported here point to an important role for novel genes in venom function, and this information should assist with the selection of candidates in future functional studies.

## Conclusions

In this study we used RNA-seq to generate a comprehensive dataset of expression information for VG and ovary in the developing model system *Nasonia*. Information on the expression level of the 79 venom protein encoding genes provides an unbiased approach for selecting RNAi candidates, especially as bioinformatic annotations are likely to be unreliable. As our knowledge of venom gene function increases our ability to understand the fundamental molecular mechanisms underlying envenomation will also be improved. This latter knowledge will be important in future efforts to use *Nasonia* venom in drug development pipelines.

## Methods

### *Nasonia* and tissue collection

The *Nasonia* (LABII strain) was a gift from Prof. Jack Werren, University of Rochester, USA. The *Nasonia* were hosted on *Lucilia sericata* pupae obtained from a commercial insectary (Biosupplies Ltd). Venom gland (VG) and ovary tissue from mated, host exposed, 1–3 day old females, was dissected under aseptic conditions and placed immediately into ice cold Trizol (Ambion). Each replicate contained VG or ovaries tissue pooled from between 40 and 50 individuals.

### RNA-seq and functional annotation

RNA was extracted using the modified RNeasy (Qiagen) protocol. Briefly, tissue was initially homogenised in 1 ml of Trizol before the addition of 0.2 ml chloroform (Merck). The sample was centrifuged at 10,000 rpm for 10 min at room temperature and the supernatant was transferred to a Qiagen RNeasy column. The column was centrifuged at 10,000 rpm for 15 s before the bound RNA was washed twice with 0.5 ml Qiagen RPE buffer. The RNA was eluted in 30 ul of RNase free water as described in the RNeasy protocol. The quality of the purified RNA was verified using a Bioanalyzer (Agilent) with all samples having a RNA integrity score >7. Poly-T beads were used to enrich for mRNA and TruSeq (Illumina) stranded cDNA sequencing libraries were constructed by the Otago Genome Service (NZGL). The 100 bp paired end sequencing run was performed on the Illumina HiSeq2500 platform.

The raw RNA-seq data was processed with fastq-mcf to remove sequencing adapters and primers [56]. The reads were quality trimmed to a Phred score of >20 using SolexaQA v3.1.3 [57]. The paired-end reads were then mapped to the *N. vitripennis* genome [4] using Tophat2 (version 2.1) with the default settings, except for library type set as “fr-firststrand” based on the sequencing chemistry used during the library preparation [58]. Read counts for gene feature in the *N. vitripennis* official gene set version 2 were generated using HTSeq-count (version 0.6.1p) with ‘union mode’ on exon features and incorporating strand information [59].

Differential expression between the ovary and VG read counts was determined using DESeq as described in the package vignette [25, 60]. A gene was considered differentially expressed if it had an adjusted *p*-value < 0.05. Principal component analysis was performed using R with the prcomp function. The heatmaps were generated using heatmap.2 (ggplots CRAN package) and clustered based on euclidean distance. A one-tailed enrichment analysis for enrichment of functional categories was carried out with Blast2GO using both the newly annotated venom and ovary expressed genes as the target and the entire gene set as the reference [61]. Soley for the purposes of ranking genes based on expression level, we divided the DESeq normalised read counts by the transcript length (in kilobases) to compensate for the effects of gene length on counts. These length normalised counts are shown in Table 2 and Table 3 and also provided in Additional files 1, 2, 3 and 4. The expression level boundaries of high, medium, and lowly expressed venom genes were determined by binning all genes into windows of expression of 100. A bin was classified as low expressed if the value of the bin was multiplied by its number of containing genes and this value was less than a third of the sum of these values, high if equal or greater than two thirds, and medium elsewise.

The Cufflinks pipeline was used to identify putative alternative transcripts as well as unannotated genes based on comparisons to the current *Nasonia* gene models as described in the package documentation [29]. Novel transcripts were annotated using Blast2GO v2.8 with the default parameters [61]. Normalised read counts were generated for the new gene models by merging the cufflinks models with those of OGS version 2 and repeating the DESeq based analysis described above.

### Metatranscriptomics

Reads that did not align with the *Nasonia* genome using Tophat2 were used in a Diamond BlastX search against the NCBI non-redundant (nr) database. Results presented do not include data from unmapped reads that were discarded by Tophat2 during the generation of non-redundant splice junctions. The high number of unmapped reads in the third VG sample necessitated that only a random sample of 10 % of the reads from this particular sample were used due to memory limitations. Megan5 was used for visualisation of the taxonomic distribution of reads in the data [42].

### Semi-quantitative PCR

Complementary DNA was generated from 1 ug of RNA extracted from dissected ovary and VG tissue using the transcriptor first strand cDNA synthesis kit (Roche) as described by the manufacturer. The PCR was then performed using primers for the housekeeping gene *RP49*: 5’-CTTCCGCAAAGTCCTTGTTC-3’, 5’-TTTATTCATTCTCCTCAGAACG-3’. The primers specific for *Nasvi2EG007167* were: 5’-TGGCTGTCATCAGATTGACG-3’, 5’-TATCCTGGAGCCAGTGTAG-3’. Reaction tubes were removed at 24 and 30 cycles, at a denaturing temperature of 94 °C for 30 s, annealing temperature of 55 °C for 30 s, and extension temperature of 72 °C for 45 s with one unit of *taq* polymerase (Roche).

## Additional files

Additional file 1: DESeq results from the VG and Ovary RNA-seq data for all *Nasonia* 2.0 gene models. (XLSX 3172 kb) Additional file 2: Sequence based clustering of venom encoding genes using BLASTClust. (DOCX 18 kb) Additional file 3: Novel venom gland and ovary transcribed regions predicted by cufflinks. (TXT 406 kb) Additional file 4: Genomic locations of 472 new gene predictions based on the ovary and VG RNA-seq data. (XLSX 50 kb) Additional file 5: DESeq based differential expression data for all *Nasonia* 2.0 gene models merged with the new gene models identified in this study. (XLSX 3636 kb)
