# Supplementary material for: The venom gland transcriptome of the parasitoid wasp Nasonia vitripennis highlights the importance of novel genes in venom function
Source: BMC Genomics. 2016 Aug 8;17:571. doi: 10.1186/s12864-016-2924-7 (PMC4977848; doi:10.1186/s12864-016-2924-7)
Supplement: Additional file 2: — Sequence based clustering of venom encoding genes using BLASTClust. (DOCX 18 kb) [file 12864_2016_2924_MOESM2_ESM.docx]

Sequence based clustering of venom protein encoding genes using BLASTClust

| Gene | Cluster^ | Annotations† |
| --- | --- | --- |
| Nasvi2EG020296  Nasvi2EG020297  Nasvi2EG020586  Nasvi2EG022916  Nasvi2EG022918  Nasvi2EG007166  Nasvi2EG007167  Nasvi2EG005749  Nasvi2EG011442  Nasvi2EG007347  Nasvi2EG022914 | 1 | Serine protease/CLIP  Serine protease/CLIP  Serine protease  Serine protease  Serine protease  Serine protease  Serine protease  Serine protease  Serine protease  Serine protease  Serine protease |
| Nasvi2EG019611  Nasvi2EG015589 | 2 | Laccase  Laccase |
| Nasvi2EG036525  Nasvi2EG008779 | 3 | Aminotransferase-like  Aminotransferase-like |
| Nasvi2EG005784  Nasvi2EG005790 | 4 | Antigen 5-like protein  Antigen-5 like |
| Nasvi2EG009661  Nasvi2EG009662 | 5 | Venom protein V  Venom protein Z |
| Nasvi2EG014069  Nasvi2EG014575 | 6 | Venom protein L  Venom protein K |
| Nasvi2EG000351  Nasvi2EG000354 | 7 | Cysteine-rich/TIL 2  Cysteine-rich/TIL 1 |
| Nasvi2EG009433 | 8 | Cysteine-rich/KU |
| Nasvi2EG008007 | 9 | Angiotensin-converting |
| Nasvi2EG023753 | 10 | Dipeptidylpeptidase IV |
| Nasvi2EG008324 | 11 | Trehalase |
| Nasvi2EG004824 | 12 | Venom protein E |
| Nasvi2EG006243 | 13 | γ-glutamyltranspeptidase |
| Nasvi2EG013885 | 14 | γ-Glutamyl transpeptidase 2 |
| Nasvi2EG010351 | 15 | Glucose dehydrogenase-like |
| Nasvi2EG016421 | 16 | α-Esterase |
| Nasvi2EG006920 | 17 | Arylsulphatase b |
| Nasvi2EG000112 | 18 | Apyrase |
| Nasvi2EG012510 | 19 | Chitinase 5 |
| Nasvi2EG009035 | 20 | Endonuclease-like |
| Nasvi2EG020295 | 21 | Serine protease/CLIP |
| Nasvi2EG002112 | 22 | β-1,3-Glucan recognition |
| Nasvi2EG007615 | 23 | Lipase |
| Nasvi2EG011463 | 24 | Inositol phosphatase |
| Nasvi2EG021024 | 25 | Serine protease homologue |
| Nasvi2EG007282 | 26 | Serine protease |
| Nasvi2EG009991 | 27 | Acid phosphatase |
| Nasvi2EG026553 | 28 | Lipoprotein receptor-like |
| Nasvi2EG037342 | 29 | Calreticulin |
| Nasvi2EG022626 | 30 | Serine protease/CUB |
| Nasvi2EG004342 | 31 | Lipase-like |
| Nasvi2EG010516 | 32 | Metalloprotease |
| Nasvi2EG021414 | 33 | Nucleoside hydrolase |
| Nasvi2EG015708 | 34 | Aspartylglucosaminidase |
| Nasvi2EG001168 | 35 | Immunoglobulin-like |
| Nasvi2EG011314 | 36 | Venom protein W |
| Nasvi2EG012285 | 37 | Acid phosphatase |
| Nasvi2EG013838 | 38 | Venom protein F |
| Nasvi2EG009664 | 39 | Cysteine-rich/Pacifastin 1 |
| Nasvi2EG000909 | 40 | C1q-like venom protein |
| Nasvi2EG004152 | 41 | Venom protein R |
| Nasvi2EG006543 | 42 | γ-Glutamyl cyclotransferase- |
| Nasvi2EG009665 | 43 | Cysteine-rich/Pacifastin 2 |
| Nasvi2EG019091 | 44 | Venom protein M |
| Nasvi2EG009647 | 45 | Venom protein Q |
| Nasvi2EG020997 | 46 | Venom protein J |
| Nasvi2EG016543 | 47 | Venom protein N |
| Nasvi2EG005645 | 48 | Venom protein O |
| Nasvi2EG004628 | 49 | Venom protein D |
| Nasvi2EG010245 | 50 | Odorant-binding protein |
| Nasvi2EG013736 | 51 | Serine protease inhibitor 1 |
| Nasvi2EG014072 | 52 | Venom protein X |
| Nasvi2EG004144 | 53 | Venom protein T |
| Nasvi2EG013868 | 54 | Venom protein Y |
| Nasvi2EG009648 | 55 | Venom protein G |
| Nasvi2EG008596 | 56 | Venom protein P |
| Nasvi2EG012348 | 57 | Venom protein H |
| Nasvi2EG037101 | 58 | Chitin binding protein-like |
| Nasvi2EG016379 | 59 | Venom protein U |
| Nasvi2EG003930 | 60 | Serine protease inhibitor 2 |
| Nasvi2EG002524 | 61 | Venom protein I |
| Nasvi2EG000667 | 62 | Serine proteinase inhibitor |
| Nasvi2EG006071 | 63 | Venom protein S |

^BLASTClust (<http://toolkit.tuebingen.mpg.de/blastclust>) output based on protein sequences of the 79 venom genes, sequence length covered set to 70% and minimum identity threshold set at 5%

†Annotations taken from De Graaf et al (2010) (reference 9 in the main manuscript)
